# Supplementary material for: Population Genetics Revealed a New Locus That Underwent Positive Selection in Barley
Source: Int J Mol Sci. 2019 Jan 8;20(1):202. doi: 10.3390/ijms20010202 (PMC6337186; doi:10.3390/ijms20010202)
Supplement: Supplementary file 1 [file ijms-20-00202-s001.pdf]

Supplementary Information

Population genetics revealed a new locus that underwent positive selection in barley

Stephan Reinert: Institute of Crop Science and Resource Conservation, Plant Breeding, University of Bonn, Katzenburgweg 5, 53115 Bonn, Germany

Alina Osthoff: Institute of Crop Science and Resource Conservation, Plant Breeding, University of Bonn, Katzenburgweg 5, 53115 Bonn, Germany

Jens Léon: Institute of Crop Science and Resource Conservation, Plant Breeding, University of Bonn, Katzenburgweg 5, 53115 Bonn, Germany

Ali Ahmad Naz*: Institute of Crop Science and Resource Conservation, Plant Breeding, University of Bonn, Katzenburgweg 5, 53115 Bonn, Germany

* Correspondence:

Ali Ahmad Naz

Tel: ++49-(0)228-73-2752, Email: a.naz@uni-bonn.de

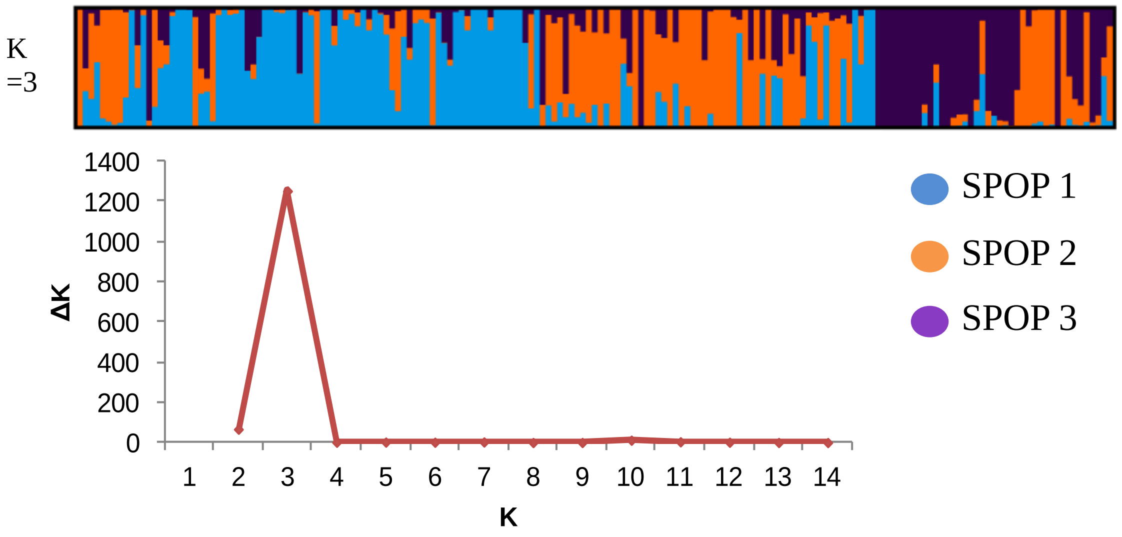

Figure S1: Population structure and genetic differentiation analysis for barley diversity panel. Population structure of 179 accessions calculated with 5892 polymorphic SNP marker revealed three sub-groups (K=3). The genetic distribution within each accession is denoted as a colored vertical line. The three different colors represent different sub-groups. Blue: SPOP 1; Orange: SPOP 2; Purple: SPOP 3.

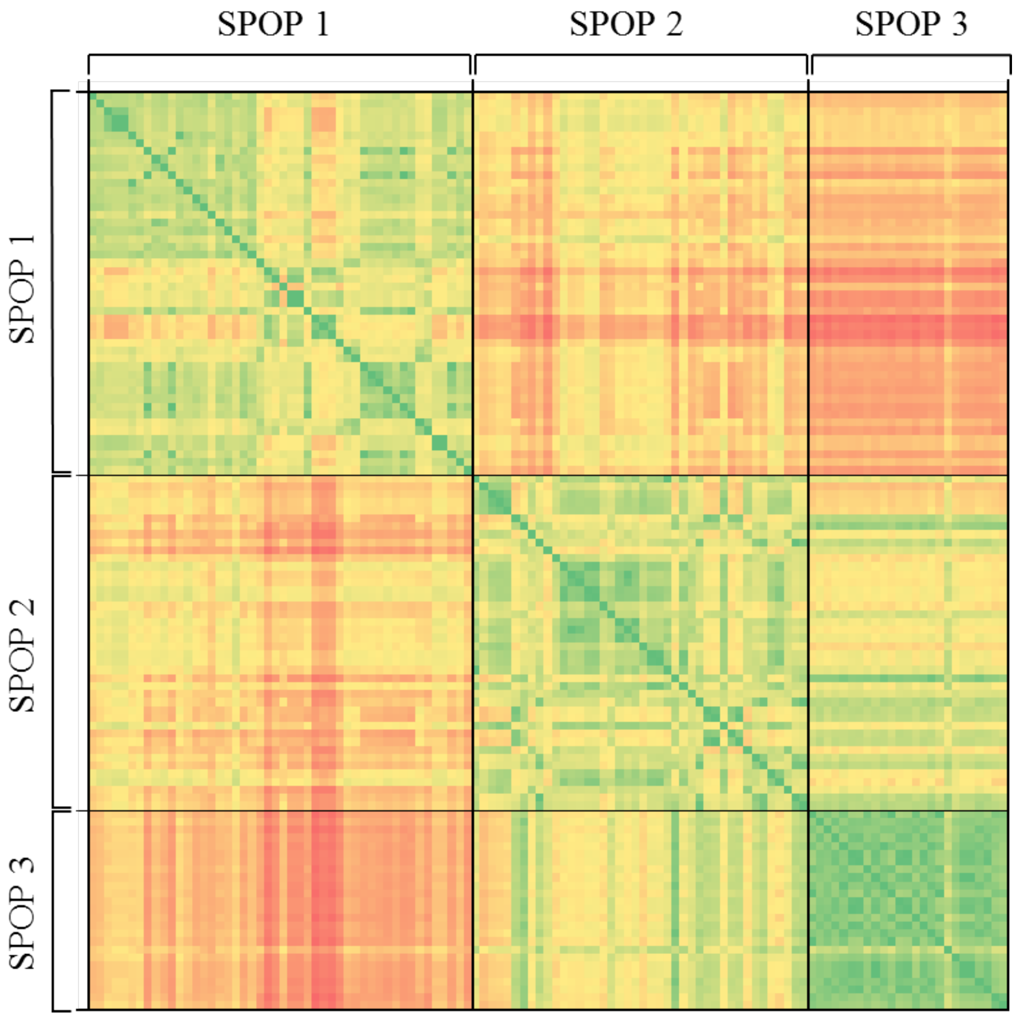

Figure S2: Genetic comparison of global genomic groups. Each group contains the genotypes sorted into sub-groups (SPOPs) based on membership coefficient ≥0.85. Dark green: Rogers distance coefficient of 1.00, dark red: Rogers distance coefficient of 0.00

Table S1: Global barley population. List of all Genotypes which are included in the global barley population with accession number, ID, sub-group membership, SNP allele, CAPS allele, country of collection, the biological status, Altitude, Longitude and Latitude.

No	Accession	ID	Sub-group	SNP	CAPS	Country	Biological status	Altitude	Longitude	Latitude
1	HOR 9721	RBC010	SPOP I	G	T	Libya	Wild type	NN 590	32°44'54" N	21°45'38" E
2	HOR 9840	RBC012	SPOP I	G	T	Libya	Wild type	NN 656	32°47'46" N	22°7'18" E
3	ICB 180006	RBC017	SPOP I	G	T	Syria	Wild type	NN 250	35°49'42" N	036°18'28"E
4	ICB 180862	RBC018	SPOP I	G	T	Syria	Wild type			
5	ICB 180902	RBC019	SPOP I	G	T	Syria	Wild type			
6	IG 121857	RBC020	SPOP I	G	T	Syria	Wild type	NN 1059	32°33'00"N	036°35'42"E
7	ICB 180092	RBC025	SPOP I	G	T	Palestine	Wild type			
8	ICB 180117	RBC026	SPOP I	G	T	Palestine	Wild type			
9	ICB 180410	RBC027	SPOP I	G	T	Palestine	Wild type			
10	ICB 180994	RBC028	SPOP I	G	T	Palestine	Wild type	NN 54	31°40'00"N	034°34'00"E
11	ICB 181160	RBC029	SPOP I	G	T	Iran	Wild type			
12	ICB 181442	RBC033	SPOP I	G	T	Jordan	Wild type	NN 782	31°17'51"N	035°50'41"E
13	ICB 181418	RBC034	SPOP I	G	T	Jordan	Wild type	NN 812	31°46'47"N	035°48'00"E
14	ICB 180013	RBC035	SPOP I	G	T	Jordan	Wild type	NN 480	32°14'25"N	035°51'55"E
15	ICB 181268	RBC036	SPOP I	G	T	Jordan	Wild type	NN750	32°18'6"N	035°55'17"E
16	ICB 180007	RBC037	SPOP I	G	T	Jordan	Wild type	NN 591	32°29'15"N	035°55'39"E
17	ICB 180260	RBC038	SPOP I	G	T	Israel	Wild type	NN 36	33°00'00"N	035°08'00"E
18	ICB 180329	RBC040	SPOP I	G	T	Israel	Wild type	NN 83	31°26'00" N	34°29'00" E
19	ICB 180508	RBC041	SPOP I	G	T	Israel	Wild type			
20	ICB 180046	RBC043	SPOP I	G	T	Iraq	Wild type	NN 323	36°00'00"N	043°31'00"E
21	ICB 180069	RBC044	SPOP I	G	T	Iraq	Wild type	NN 470	34°48'00"N	045°36'00"E
22	HOR 2514	RBC046	SPOP I	G	T	India	Wild type			
23	HOR 11421	RBC047	SPOP I	G	T	India	Landrace	NN 2880	31°41'31" N	77°31'35" E
24	HOR 8367	RBC048	SPOP I	G	T	India	Landrace			
25	HOR 8372	RBC049	SPOP I	G	T	India	Landrace			
26	HOR 7603	RBC050	SPOP I	G	T	Pakistan	Landrace	NN 2830	36°05'40" N	074°04'35" E
27	HOR 7599	RBC052	SPOP I	G	T	Pakistan	Landrace	NN 2100	36°17'40" N	073°46'57" E
28	ICB 181243	RBC053	SPOP I	G	T	Pakistan	Wild type	NN 1560	30°18'00" N	066°54'00" E
29	HOR 1479	RBC059	SPOP I	G	T	China	Landrace	NN 3685	29°21'00" N	090°39'00" E
30	HOR 1510	RBC060	SPOP I	G	T	China	Landrace	NN 3650	29°38'59" N	091°05'59" E
31	HOR 1566	RBC061	SPOP I	G	T	China	Landrace	NN 4076	29°15'19" N	090°49'59" E
32	NGB4668	RBC063	SPOP I	G	T	Afghanistan	Landrace			
33	NGB6952	RBC066	SPOP I	G	T	Afghanistan	Landrace			
34	NGB9599	RBC067	SPOP I	G	T	Afghanistan	Landrace			
35	ICB 181498	RBC069	SPOP I	G	T	Uzbekistan	Wild type	NN 350	41°9'58"N	069°02'00"E
36	IG 124000	RBC070	SPOP I	G	T	Uzbekistan	Wild type	NN 1450	39°42'00"N	068°02'45"E
37	IG 124017	RBC071	SPOP I	G	T	Uzbekistan	Wild type	NN 700	40°00'00"N	067°05'15"E
38	ICB 180211	RBC073	SPOP I	G	T	Turkmenistan	Wild type	NN 1530	37°42'59" N	058°24'50" E

39	ICB 180215	RBC074	SPOP I	G	T	Turkmenistan	Wild type			
40	ICB 180217	RBC075	SPOP I	G	T	Turkmenistan	Wild type	NN 250	37°40'00"N	065°35'00" E
41	ICB 181492	RBC076	SPOP I	G	T	Turkmenistan	Wild type	NN 456	38°02'00"N	058°00'00"E
42	HOR 18647	RBC077	SPOP I	G	T	Japan	Landrace			
43	HOR 19848	RBC080	SPOP I	G	T	Japan	Landrace			
44	HOR 11017	RBC127	SPOP I	G	T	Greece	Wild type	NN 20	35°30'59" N	024°01'59" E
45	HOR 12418	RBC130	SPOP I	G	T	Greece	Wild type	NN 20	35°30'59" N	024°01'59" E
46	ICB 181500	RBC135	SPOP I	G	T	Tadjikistan	Wild type	NN 1030	39°28'25"N	067°30'1"E
47	ICB 180070	RBC137	SPOP I	G	T	Turkey	Wild type	NN 840	39°39'52"N	031°9'40"E
48	ICB 181162	RBC138	SPOP I	G	T	Iran	Wild type			
49	HOR 16097	RBC001	SPOP II	G	T	Egypt	Cultivar			
50	BCC 126	RBC005	SPOP II	G	T	Marocco	Landrace			
51	BCC 149	RBC006	SPOP II	G	T	Marocco	Landrace			
52	HOR 13412	RBC007	SPOP II	G	T	Marocco	Landrace	NN 596	31°22'00" N	008°31'00" W
53	BCC 131	RBC008	SPOP II	G	T	Marocco	Landrace			
54	HOR 930	RBC021	SPOP II	A	-T	Turkey	Landrace			
55	HOR 19883	RBC024	SPOP II	A	-T	Turkey	Landrace			
56	HOR 20921	RBC042	SPOP II	A	-T	Israel	Landrace			
57	HOR 17616	RBC056	SPOP II	A	-T	Nepal	Landrace			
58	HOR 56	RBC062	SPOP II	A	-T	China	Landrace			
59	HOR 4124	RBC089	SPOP II	A	-T	Mexico	Landrace			
60	BCC 848	RBC091	SPOP II	G	T	Mexico	Cultivar			
61	HOR 7443	RBC093	SPOP II	A	-T	Bolivia	Landrace			
62	HOR 7446	RBC094	SPOP II	R	T/-T	Bolivia	Landrace			
63	HOR 2981	RBC097	SPOP II	G	T	Chile	Cultivar			
64	HOR 20110	RBC099	SPOP II	G	T	Chile	Landrace			
65	HOR 14485	RBC100	SPOP II	G	T	Chile	Landrace			
66	HOR 10843	RBC103	SPOP II	G	T	Colombia	Landrace	NN 2000	5°45'15" N	73°34'37" W
67	BCC 927	RBC105	SPOP II	A	-T	Peru	Cultivar			
68	HOR 7449	RBC107	SPOP II	A	-T	Peru	Landrace			
69	HOR 9565	RBC108	SPOP II	A	-T	Peru	Landrace			
70	HOR 17307	RBC110	SPOP II	G	T	Uruguay	Landrace			
71	BCC 862	RBC111	SPOP II	G	T	Uruguay	Cultivar			
72	BCC 896	RBC112	SPOP II	G	T	Uruguay	Cultivar			
73	HOR 35	RBC113	SPOP II	G	T	Australia	Cultivar			
74	HOR 4206	RBC114	SPOP II	A	-T	Australia	Cultivar			
75	HOR 18209	RBC116	SPOP II	G	T	Australia	Cultivar			
76	HOR 13965	RBC118	SPOP II	A	-T	Australia	Cultivar			
77	BCC 1551	RBC120	SPOP II	A	-T	Armenia	Cultivar			
78	BCC 1474	RBC123	SPOP II	A	-T	Ukraine	Cultivar			
79	BCC 1505	RBC125	SPOP II	A	-T	Ukraine	Cultivar			
80	HOR 1131	RBC129	SPOP II	G	T	Greece	Landrace	NN 226	35°27'52" N	023°46'17" E
81	HOR 199	RBC131	SPOP II	A	-T	Russia	Landrace			

82	HOR 3372	RBC132	SPOP II	A	-T	Russia	Landrace			
83	BCC 1348	RBC164	SPOP II	G	T/-T	Spain	Cultivar			
84	BCC 1523	RBC165	SPOP II	A	-T	Spain	Cultivar			
85	HOR 19267	RBC166	SPOP II	A	-T	Spain	Landrace			
86	BCC 1586	RBC167	SPOP II	G	T/-T	Spain	Cultivar			
87	HOR 873	RBC168	SPOP II	G	-T	France	Cultivar			
88	HOR 1132	RBC169	SPOP II	G	T/-T	France	Landrace	NN 650m	42°27'55" N	2°54'49" E
89	HOR 11790	RBC171	SPOP II	A	-T	France	Cultivar			
90	HOR 12047	RBC175	SPOP II	A	-T	GB/Irland	Landrace	NN 157	52°24'28" N	001°56'41" W
91	HOR 16287	RBC013	SPOP III	A	-T	Sudan	Landrace			
92	BCC 871	RBC098	SPOP III	A	-T	Chile	Cultivar			
93	CCS 004	RBC139	SPOP III	A	-T	Germany	Cultivar			
94	CCS 010	RBC140	SPOP III	A	-T	Germany	Cultivar			
95	CCS 012	RBC141	SPOP III	A	-T	Germany	Cultivar			
96	CCS 018	RBC142	SPOP III	A	-T	Germany	Cultivar			
97	CCS 023	RBC143	SPOP III	A	-T	Germany	Cultivar			
98	CCS 041	RBC144	SPOP III	A	-T	Germany	Cultivar			
99	CCS 052	RBC145	SPOP III	A	-T	Germany	Cultivar			
100	CCS 060	RBC146	SPOP III	A	-T	Germany	Cultivar			
101	CCS 081	RBC148	SPOP III	A	-T	Germany	Cultivar			
102	CCS 084	RBC150	SPOP III	A	-T	Germany	Cultivar			
103	CCS 086	RBC151	SPOP III	A	-T	Germany	Cultivar			
104	CCS 089	RBC152	SPOP III	A	-T	Germany	Cultivar			
105	CCS 095	RBC153	SPOP III	A	-T	Germany	Cultivar			
106	CCS 096	RBC154	SPOP III	A	-T	Germany	Cultivar			
107	CCS 109	RBC155	SPOP III	A	-T	Germany	Cultivar			
108	Agueda	RBC158	SPOP III	A	-T	Germany	Cultivar			
109	Montoya	RBC159	SPOP III	A	-T	Germany	Cultivar			
110	Danielle	RBC160	SPOP III	A	-T	Germany	Cultivar			
111	Britney	RBC161	SPOP III	A	-T	Germany	Cultivar			
112	Andreia	RBC162	SPOP III	A	-T	Germany	Cultivar			
113	BCC 1380	RBC170	SPOP III	A	-T	France	Cultivar			
114	NGB8822	RBC176	SPOP III	A	-T	GB/Irland	Landrace			
115	NGB9480	RBC177	SPOP III	A	-T	GB/Irland	Landrace			
	Scarlett	Control 1			-T	Germany	Cultivar			
	ISR42-8	Control 2			T	Israel	Wild type			

Sub-group = Membership to a sub-group (SPOP I, SPOP II, SPOP III) based on the membership coefficient of ≥0.85, otherwise grouped in ADMIX. SNP = Allele of marker SCRI_RS_170235 for particular genotype; Alleles: G = guanine (Major allele), A = adenine (Minor allele), R = heterozygous / heterogeneous, "-" = missing value. CAPS = Allele of CAPS marker for particular genotype; Alleles: T = thymine present, -T = thymine deletion, T/-T = heterozygous / heterogeneous, "-" = missing value.

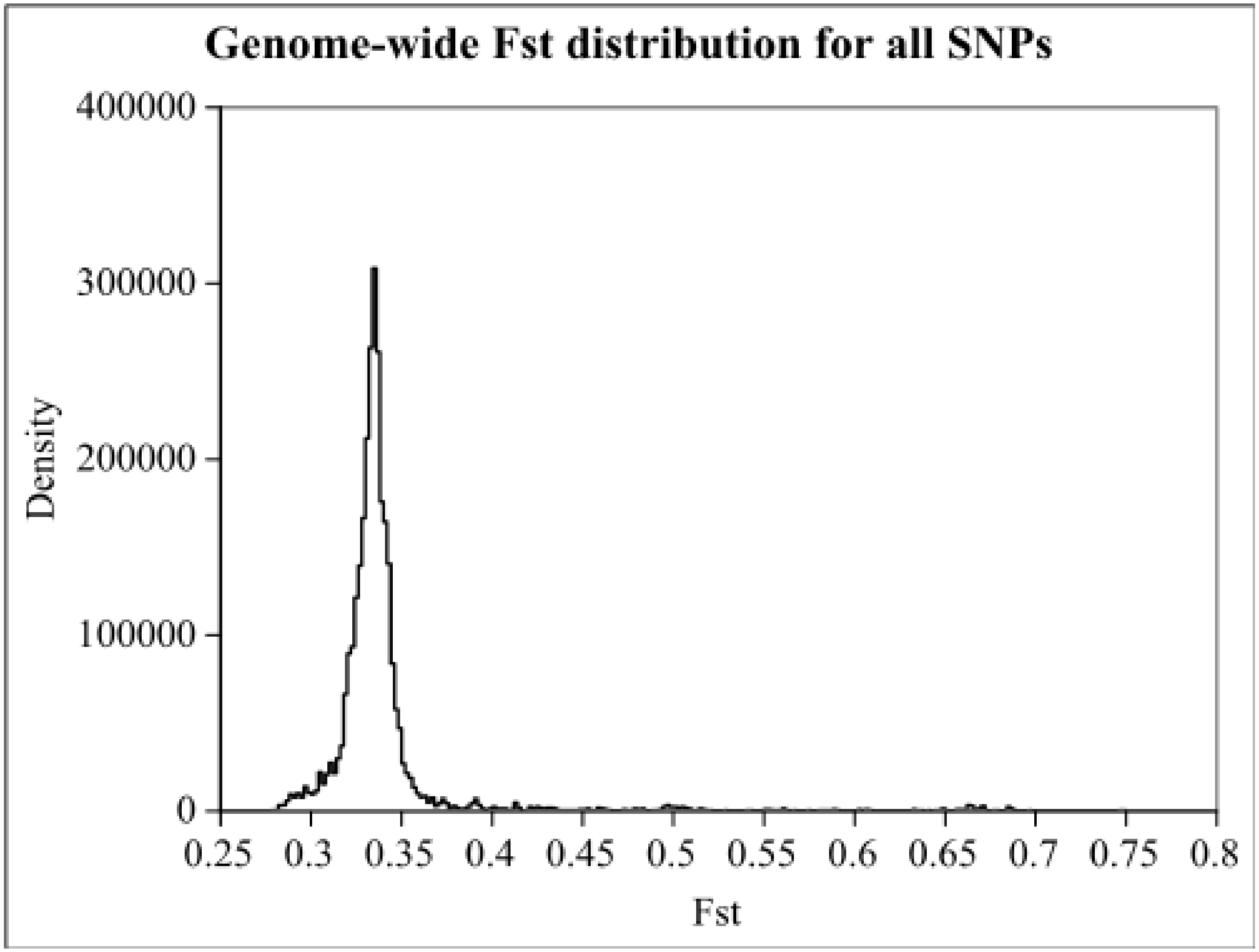

Figure S3. Fst-distribution plot for all SNP markers in barley diversity set.

Table S2. Distribution of point mutation among the genotypes and the sub-populations.

	WT	L	C	**Total**	% -T	% T	% T/-T
SPOP 1	35	13	0	**48**	0.00	100.00	0.00
SPOP 2	0	24	18	**42**	52.38	38.10	9.52
SPOP 3	0	3	22	**25**	100.00	0.00	0.00
**Total**	**35**	**40**	**40**	**115**			
% -T	0.00	40.00	77.50	55.65			
% T	100.00	55.00	17.50	40.87			
% T/-T	0.00	5.00	5.00	3.48			

WT: Wild accession, L: Landrace, C: Modern cultivar, % -T: Percentage of genotypes missing thymine within CDS of CASPL4 gene, % T: Percentage of genotypes carrying an additional thymine within CDS of CASPL4 gene. %T/-T: Percentage of heterozygous genotypes.

Table S3. List of primer for CAPS analysis. TA: Annealing temperature in degrees Celcius

**Primer**	**TA°C**	**Sequence**	**Fragment (bp)**	**Experiment**	**Note**
*Hv*CAPS002-fwd	56.0	AACCGATGACAAACGCCAC	302	Outlier	Frag 1: 75 bp, Frag 2: 227 bp
*Hv*CAPS002-rev	56.0	CCACGCCAAGCCTCTAAAG			

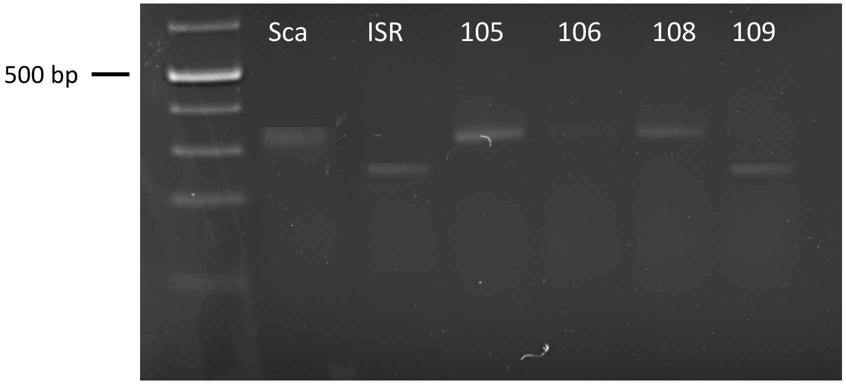

Figure S4. Selection of introgression line IL S42IL-109 carrying wild alleles of candidate gene in the cultivated background. Restriction digest of PCR fragments with AvaII. Genotypes with the 305 bp fragment carrying a 1 bp deletion. Genotypes with the 230 bp fragment carrying an additional thymine which leaded to an AvaII restriction site. Sca= Scarlett; ISR= ISR42-8; 105= S42IL105; 106= S42IL106; 108= S42IL108; 109= S42IL109.

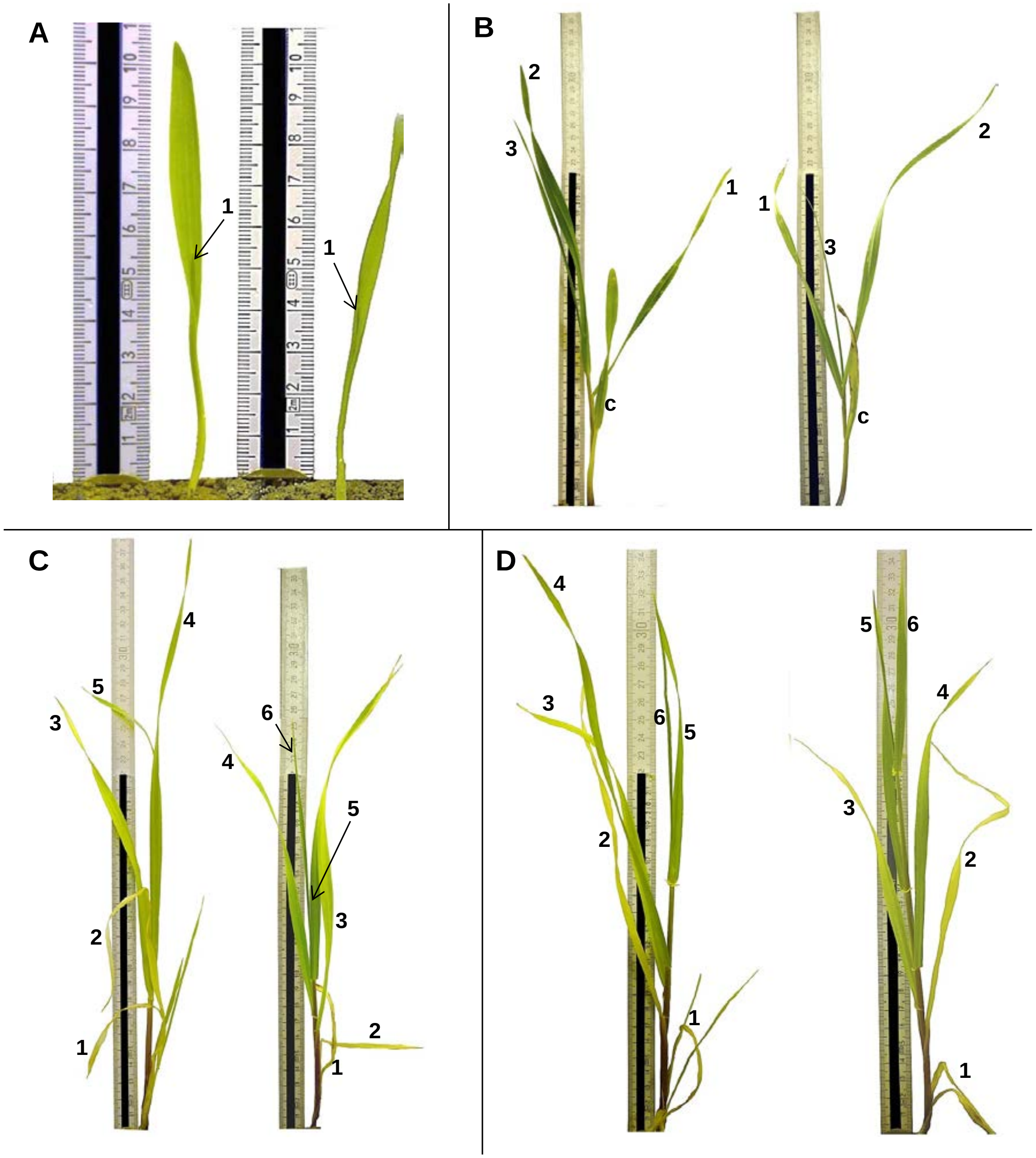

Figure S5: Comparison of seedling development between Scarlett on the left and S42IL-109 on the right side. A) five days after sowing: Beginning of phyllochron 1 (first leaf is visible). B) 18 days after sowing: Middle of phyllochron 3 (3rd leaf developed but 4th leaf tip not visible yet). C) 31 days after sowing: Middle of phyllochron 5 in Scarlett (5th leaf developed), end of phyllochron 5 in S42IL-109 (leaf tip of 6th leaf visible). D) 38 days after sowing: End of phyllochron 5 in both genotypes (6th leaf visible)
